# Gadoxetic acid uptake as a molecular imaging biomarker for sorafenib resistance in patients with hepatocellular carcinoma: a post hoc analysis of the SORAMIC trial

**DOI:** 10.1007/s00432-021-03803-3

**Published:** 2021-09-20

**Authors:** Osman Öcal, Daniel Rössler, Antonio Gasbarrini, Thomas Berg, Heinz-Josef Klümpen, Irene Bargellini, Bora Peynircioglu, Otto van Delden, Christian Schulz, Kerstin Schütte, Roberto Iezzi, Maciej Pech, Peter Malfertheiner, Bruno Sangro, Jens Ricke, Max Seidensticker

**Affiliations:** 1 Department of Radiology, University Hospital, Ludwig Maximilian University of Munich, Marchioninistrasse 15, 81377 Munich, Germany; 2 Department of Medicine II, University Hospital, LMU Munich, Munich, Germany; 3 Fondazione Policlinico Gemelli IRCCS, Università’ Cattolica del Sacro Cuore, Roma, Italy; 4 Klinik Und Poliklinik Für Gastroenterologie, Sektion Hepatologie, Universitätsklinikum Leipzig, Leipzig, Germany; 5 Department of Medical Oncology, Amsterdam University Medical Centers, University of Amsterdam, Amsterdam, The Netherlands; 6 Department of Vascular and Interventional Radiology, University Hospital of Pisa, Pisa, Italy; 7 Department of Radiology, Hacettepe University, Ankara, Turkey; 8 Department of Radiology and Nuclear Medicine, Academic Medical Center, University of Amsterdam, Amsterdam, The Netherlands; 9 Department of Internal Medicine and Gastroenterology, Niels-Stensen-Kliniken Marienhospital, Osnabrück, Germany; 10 Department of Gastroenterology, Hepatology and Infectious Diseases, Otto-Von-Guericke University, Magdeburg, Germany; 11 Fondazione Policlinico Universitario A. Gemelli IRCCS, UOC Di Radiologia, Dipartimento Di Diagnostica Per Immagini, Radioterapia Oncologica ed Ematologia, Roma, Italy; 12 Departments of Radiology and Nuclear Medicine, University of Magdeburg, Magdeburg, Germany; 13 Liver Unit, Clínica Universidad de Navarra-IDISNA and CIBEREHD, Pamplona, Spain

**Keywords:** Hepatocellular carcinoma, Sorafenib, Radioembolization, Gadoxetic acid, WNT, ß-Catenin

## Abstract

**Purpose:**

Gadoxetic acid uptake on hepatobiliary phase MRI has been shown to correlate with ß-catenin mutation in patients with HCC, which is associated with resistance to certain therapies. This study aimed to evaluate the prognostic value of gadoxetic acid uptake on hepatobiliary phase MRI in patients with advanced HCC receiving sorafenib.

**Methods:**

312 patients with available baseline hepatobiliary phase MRI images received sorafenib alone or following selective internal radiation therapy (SIRT) within SORAMIC trial. The signal intensity of index tumor and normal liver parenchyma were measured on the native and hepatobiliary phase MRI images, and relative tumor enhancement higher than relative liver enhancement were accepted as high gadoxetic acid uptake, and its prognostic value was assessed using univariate and multivariate Cox proportional hazard models.

**Results:**

The median OS of the study population was 13.4 (11.8–14.5) months. High gadoxetic acid uptake was seen in 51 (16.3%) patients, and none of the baseline characteristics was associated with high uptake.

In univariate analysis, high gadoxetic acid uptake was significantly associated with shorter overall survival (10.7 vs. 14.0 months, *p* = 0.005). Multivariate analysis confirmed independent prognostic value of high gadoxetic acid uptake (HR, 1.7 [1.21–2.3], *p* = 0.002), as well as Child–Pugh class (*p* = 0.033), tumor diameter (*p* = 0.002), and ALBI grade (*p* = 0.015).

**Conclusion:**

In advanced HCC patients receiving sorafenib (alone or combined with SIRT), high gadoxetic acid uptake of the tumor on pretreatment MRI, a surrogate of ß-catenin mutation, correlates with shorter survival. Gadoxetic acid uptake status might serve in treatment decision-making process.

**Supplementary Information:**

The online version contains supplementary material available at 10.1007/s00432-021-03803-3.

## Introduction

The global incidence and mortality of liver cancer continue to increase, with almost one million new cases diagnosed in 2017 (Lin et al. 2020). A large proportion of patients with hepatocellular carcinoma are diagnosed at a late stage and thus are not eligible for potentially curative therapies. As there has been considerable progress in systemic therapy in recent years, treatment selection has become more challenging for clinicians. After almost a decade of negative phase III trials following the breakthrough of the SHARP trial in 2007 (Llovet et al. 2008), a total of five new drugs have proven effective (Bruix et al. 2017; Abou-Alfa et al. 2018; Kudo et al. 2018; Zhu et al. 2019). With several options for first- and second-line therapy available in the market, there is an urgent need to identify markers for treatment benefit and guide treatment choice. The recently published IMbrave150 trial, which led to the approval of Atezolizumab in combination with Bevacizumab by the EMA and FDA, showed clear superiority of this combination over sorafenib (Finn et al. 2020). However, even the improved objective response rate of 27% leaves a considerable proportion of patients who might benefit from other treatment options. So far, the only example of biomarker-based patient selection in clinical practice is identifying a subgroup responding to Ramucirumab when this drug showed no significant effect on the overall population of the REACH trial (Zhu et al. 2015). The subsequent study REACH II showed that this VEGF inhibitor is effective in HCC patients with an AFP level of over 400 ng/ml (Zhu et al. 2019). Attempts to identify similar markers for tyrosine kinase or immune checkpoint inhibitors have been unsuccessful, with one of the best-known examples being the negative phase III trial of Tivantinib in patients with high c-Met expression in tumor tissue (Rimassa et al. 2018).

The action mechanism of immune checkpoint inhibitors has underlined the prognostic value of the immunogenic status of HCC. Previous studies have shown that some tumors are resistant by suppressing the recruitment of CD8 T cells into the tumor tissues (“immune escape”, “cold tumor”). A mouse model of HCC has identified the β-catenin pathway as the immune escape mechanism by defective recruitment of dendritic cells and impaired T-cell activity (Ruiz de Galarreta et al. 2019). Clinical implications of these findings have been identified by Harding et al. in HCC patients treated with immune checkpoint inhibitors (Harding et al. 2019). The mutation status of the patients was analyzed by next-generation sequencing (NGS), and the authors were able to show an association between alterations in WNT/β-catenin signaling and a shorter PFS in patients receiving immune checkpoint inhibitors. However, this correlation with decreased PFS and OS was not seen in patients receiving sorafenib in the same study, which led to the proposition that WNT pathway alterations might be a possible predictive biomarker for patient selection (Kudo 2020a).

Gadoxetic acid is a hepatocellular specific MRI contrast agent showing selective hepatocyte uptake, which peaks on the hepatobiliary phase, approximately 20 min after injection (Motosugi et al. 2009). Previous studies have identified organic anion transporting polypeptide 1B3 (OATP1B3) as the transporter of gadoxetic acid into the hepatocytes, as well as HCCs (Narita et al. 2009; Kitao et al. 2010; Yamashita et al. 2014). Additionally, the expression of OATP1B3 has been shown to strongly correlate with the activation of WNT/β-catenin signaling (Kitao et al. 2015). Thus, gadoxetic acid has been suggested as a potential imaging marker of the WNT/β-catenin pathway, and Ueno et al. described that gadoxetic acid uptake of tumor has the sensitivity and specificity of around 80% to predict the presence of WNT/β-catenin mutation (Ueno et al. 2014).

In the palliative arm of the SORAMIC trial (SORAfenib in combination with local MICro-therapy guided by gadolinium-EOB-DTPA-enhanced MRI, EudraCT 2009-012,576-27, NCT01126645), patients with intermediate or advanced HCC were randomized to sorafenib treatment either as monotherapy or following selective internal radiation therapy (SIRT) (Ricke et al. 2019). Gadoxetic acid-enhanced liver MRI at baseline was part of the study protocol. This post hoc analysis of the SORAMIC trial aimed to explore the potential value of gadoxetic acid uptake of HCC lesions as an imaging biomarker in patients receiving sorafenib.

## Materials and methods

### Study design and patient population

This study was a post hoc analysis of the patients who received sorafenib (alone or following SIRT) within the palliative arm of the SORAMIC trial. Results of the SORAMIC trial have been described previously (Ricke et al. 2019). SORAMIC was conducted in 38 centers from 12 countries. The study protocol was approved by each institutional review board, and all patients gave written informed consent.

In the palliative arm of the study, patients with a diagnosis of HCC in the intermediate stage (BCLC B, not eligible for TACE) or the advanced stage (BCLC C) were randomized in 11:10 ratio to either SIRT followed by sorafenib (combination arm) or sorafenib monotherapy. The main inclusion criteria were age between 18 and 85, preserved liver function (Child–Pugh scores A–B7), an Eastern Cooperative Oncology Group performance status (ECOG-PS) ≤ 2. Patients with the extrahepatic disease were not excluded as long as the disease was liver-dominant and the lungs were not involved. Inclusion into this post hoc analysis required availability of the baseline hepatobiliary phase MRI images for the centralized image analysis.

**Fig. 1:**
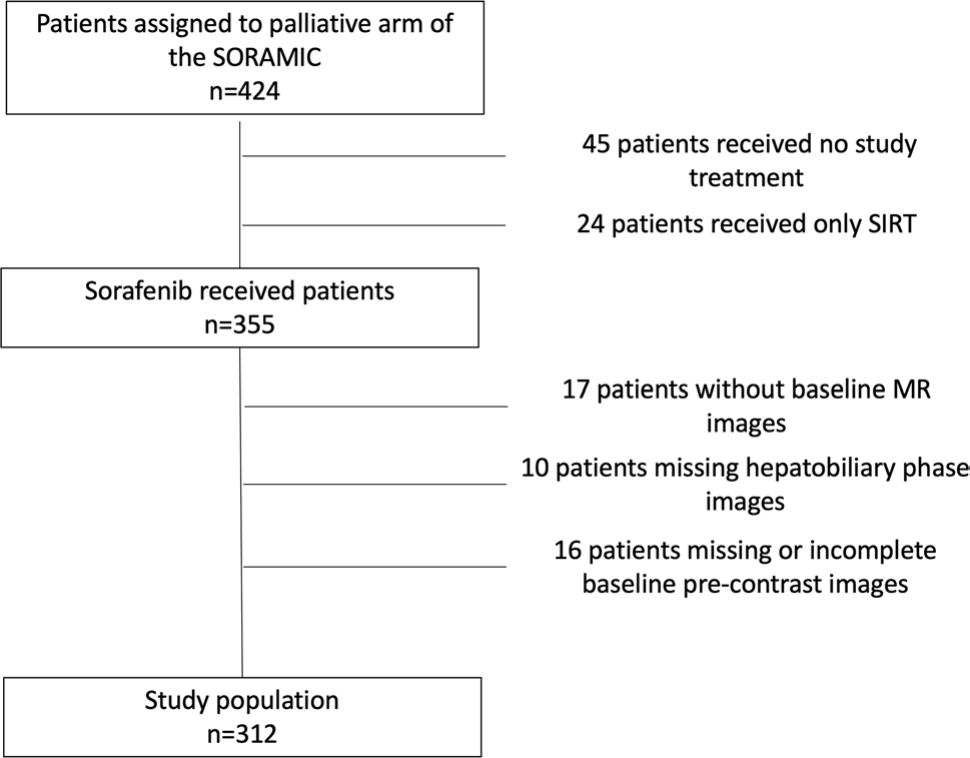
CONSORT diagram

Of the 424 patients were recruited to the palliative arm of the SORAMIC, 355 received sorafenib within the trial. While baseline MRI images were not available for 17 patients, 10 patients were excluded due to missing hepatobiliary phase images, and 16 patients were excluded due to missing or incomplete pre-contrast images. The remaining 312 patients (276 male) with a median age of 66 (41–84) years comprised the study protocol (shown in Fig. 1). Of these 312 patients, 138 (44.2%) patients randomized to combination arm, 174 (55.8%) to sorafenib arm.

### Treatment protocol

Sorafenib was started with a dose of 200 mg b.i.d. for a week, which was increased to the target dose of 400 mg b.i.d., if tolerated. In case of toxicity, dose reductions were made according to pre-defined dosing guidelines until the lowest accepted dose, 200 mg b.i.d. on alternate days. After resolution of toxicities, the dose was re-escalated stepwise to the highest tolerable dose. In patients randomized to combination therapy, SIRT therapy was performed in a lobar fashion, and sorafenib was started three days after the therapy of diseased lobe in patients with single lobe involvement. In patients with bilobar disease, treatment of the disease dominant liver lobe was followed by the contralateral lobe treatment after 4–6 weeks interval, and patients were started with sorafenib three days after the last SIRT session.

### Imaging protocol and image analysis

Imaging was performed according to a standardized MRI protocol within the diagnostic arm of the SORAMIC trial (Ricke et al. 2020). This protocol included 3D T1-weighted gradient echo (GRE) sequences obtained before (pre-contrast) and 20 min after the injection of 0.1 ml/kg gadoxetic acid (hepatobiliary phase).

Within the diagnostic arm of the SORAMIC trial, all pretreatment images were centrally assessed by two reader groups, and lesions were marked. The largest lesion chosen by the readers were further assessed for this substudy by a board-certified radiologist specialized in liver imaging blinded to all clinical information. Signal intensity (SI) of the largest lesion was measured in the slice where the tumor has the largest diameter with the largest circular ROI confined within the lesion but excluding major necrosis areas. After this, SI of the left and right liver lobe (either in the anterior or posterior sector) was measured using three different circular ROIs for each, and average SI was recorded for each lobe. Liver SI was calculated by averaging the left and right liver lobe. In patients with replacement of whole left liver lobe with tumor, measurements were done using anterior and posterior sectors separately. Measurements were repeated at the same slice and location on the pre-contrast images. Relative tumor enhancement (RTE) and relative liver enhancement (RLE) were calculated using the following formulas, and in patients with RTE higher than RLE, lesions were recorded as having high gadoxetic acid uptake:

RTE = $$\left\{\frac{{SI}_{post} of the tumor - {SI}_{pre} of the tumor }{{SI}_{pre} of the tumor}\right\},$$

RLE = $$\left\{\frac{{SI}_{post} of the liver - {SI}_{pre} of the liver }{{SI}_{pre} of the liver}\right\}.$$

In patients with follow-up CT or MRI images available for centralized image analysis, these images were evaluated according to modified Response Evaluation Criteria in Solid Tumors (mRECIST) by a board-certified radiologist blinded to all the clinical information.

### Statistical analysis

All statistical analyses were performed using R statistical and computing software, version 3.5.0 (http://www.r-project.org). Categorical variables were reported as counts and percentages, and continuous variables as means and standard deviations. Correlations were evaluated with chi-squared and Fisher’s exact tests. The Kaplan–Meier method was used to estimate the overall survival and progression-free survival of patients with high and low gadoxetic acid uptake, and survival of each group were compared using the log rank statistic. A *P* value of less than 0.05 was considered as statistically significant. Cox regression models were used to assess the effects of cofounding factors on overall survival. Variables with a *p* value of less than 0.1 in the univariate analyses were analyzed in multivariate Cox regression models to explore prognostic factors of overall survival.

## Results

### Baseline characteristics

At the end of the study, 264 (84.6%) patients were deceased, and the median OS of the study population was 13.4 (95% CI, 11.8–14.5) months. Baseline characteristics of the study population are summarized in Table 1. Of 312 patients, 212 (67.9%) had advanced (BCLC C) HCC. While 250 (80.1%) patients had underlying liver cirrhosis, 289 (92.6%) patients had Child–Pugh A, and 153 (49.0%) patients had ALBI grade 1 liver function. Image analysis revealed high gadoxetic acid uptake in the index lesion of 51 (16.3%) patients. There was no significant difference in baseline characteristics of the patients with high or low gadoxetic acid uptake.

**Table 1:** Baseline characteristics of study population

	All cohort (*n* = 312)	RTE high (*n* = 51)	RTE low (*n* = 261)	*p*
*Treatment arm*				
SIRT (+ Sorafenib)	138 (44.2%)	17 (33.3%)	121 (46.4%)	0.086
Sorafenib	174 (55.8%)	34 (66.7%)	140 (53.6%)	
Gender (male)	276 (88.4%)	43 (84.3%)	233 (89.2%)	0.310
Age (> 65 years)	147 (47.1%)	23 (45.1%)	124 (47.5%)	0.752
*ECOG-PS*				
0	217 (69.5%)	32 (62.7%)	185 (70.9%)	0.201
1&2	92 (29.5%)	19 (37.3%)	73 (28.0%)	
Missing	3 (0.9%)	–	3 (1.1%)	
Liver cirrhosis (yes)	250 (80.1%)	43 (84.3%)	207 (79.3%)	0.412
Hepatitis B	33 (10.5%)	7 (13.7%)	26 (9.9%)	0.424
Hepatitis C	73 (23.3%)	15 (29.4%)	58 (22.2%)	0.267
Alcoholic liver disease	133 (42.6%)	21 (41.1%)	112 (42.9%)	0.818
Lesion diameter > 65 mm	87 (27.8%)	17 (33.3%)	70 (26.8%)	0.342
Portal vein infiltration	131 (42.0%)	21 (41.2%)	110 (42.1%)	0.897
Extrahepatic spread	65 (20.8%)	13 (25.4%)	52 (19.9%)	0.370
*Child–Pugh*				
A	289 (92.6%)	47 (92.1%)	242 (92.7%)	0.776
B	23 (7.4%)	4 (7.9%)	19 (7.3%)	
*BCLC*				
A&B	100 (32.1%)	15 (29.4%)	85 (32.6%)	0.658
C	212 (67.9%)	36 (70.6%)	176 (67.4%)	
AFP ≥ 400	113 (36.2%)	17 (33.3%)	96 (36.7%)	0.495
*ALBI grade*				0.282
1	153 (49.0%)	22 (43.1%)	131 (50.1%)	
2	153 (49.0%)	29 (56.9%)	124 (47.5%)	
Missing	6 (1.9%)	–	6 (2.3%)	

*AFP* alfa-fetoprotein, *ALBI* albumin–bilirubin, *BCLC* Barcelona Clinic Liver Cancer, *CI* confidence interval, *ECOG-PS* Eastern Cooperative Oncology Group Performance Score, *HR* hazard ratio, *PVI* portal vein invasion

### Survival analysis

**Fig. 2:**
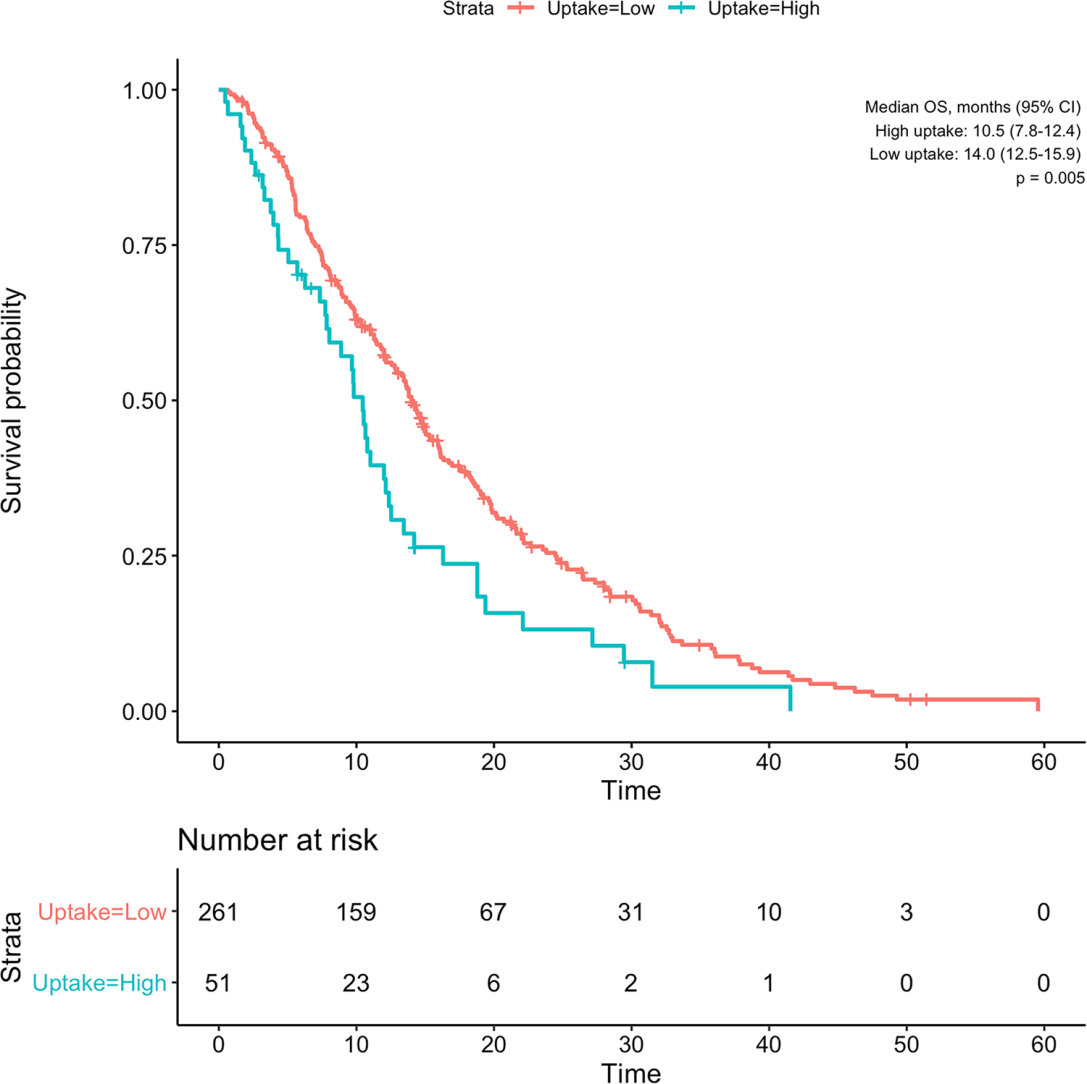
Kaplan–Meier curve showing the overall survival of patients grouped by high and low gadoxetic acid uptake

As in the main study, there was no significant difference in survival between patients received SIRT plus sorafenib and sorafenib (*p* = 0.222). Patients with high gadoxetic acid uptake had significantly shorter overall survival than patients with low gadoxetic acid uptake (10.7 vs. 14.0 months, HR, 1.6 [95% CI, 1.1–2.2]; *p* = 0.005, shown in Fig. 2). Besides this, Child–Pugh grade B (HR, 3 [95% CI, 1.9–4.8]; *p* < 0.001), ALBI grade 2 (HR, 1.7 [95% CI, 1.3–2.2]; *p* < 0.001), and tumor diameter larger than 65 mm (HR, 1.5 [95% CI, 1.2–2]; *p* = 0.003) were associated with shorter overall survival (Table 2). Additionally, although the difference was marginally non-significant, patients with ECOG-PS ≥ 1 had shorter overall survival (HR, 1.3 [95% CI, 0.98–1.7]; *p* = 0.065).

**Table 2:** Univariable and multivariable analyses of factors associated with overall survival

Parameter	Univariate analysis		Multivariate analysis	
	HR (95% CI)	*p* value	HR (95% CI)	*p* value
Treatment arm	1.2 (0.91–1.5)	0.222		
High contrast uptake	1.6 (1.1–2.2)	**0.005**	1.7 (1.21–2.3)	**0.002**
Sex (male)	0.91 (0.62–1.3)	0.622		
Age (≥ 65 years)	1.2 (0.95–1.6)	0.114		
ECOG-PS ≥ 1	1.3 (0.98–1.7)	0.069	1.2 (0.88–1.5)	0.3
Cirrhosis (yes vs. no)	1.3 (0.93–1.7)	0.135		
Hepatitis B etiology (yes)	1.2 (0.77–1.7)	0.49		
Hepatitis C etiology (yes)	1.1 (0.8–1.4)	0.628		
Alcohol etiology (yes)	0.97 (0.76–1.2)	0.825		
Metastasis (yes)	1.2 (0.89–1.6)	0.247		
PVI (yes)	1.1 (0.89–1.4)	0.324		
Child–Pugh class B	3 (1.9–4.8)	** < 0.001**	2.8 (1.75–2.6)	** < 0.001**
BCLC grade C	1.1 (0.88–1.5)	0.316		
Diameter (> 65 mm)	1.5 (1.2–2)	**0.003**	1.6 (1.18–2.1)	**0.002**
ALBI (grade ≥ 2)	1.7 (1.3–2.2)	** < 0.001**	1.6 (1.25–2.1)	** < 0.001**
AFP (≥ 400 ng/mL)	1.2 (0.94–1.6)	0.147		

Bold type indicates statistical significance

*AFP* alfa-fetoprotein, *ALBI* albumin–bilirubin, *BCLC* Barcelona Clinic Liver Cancer, *CI* confidence interval, *ECOG-PS* Eastern Cooperative Oncology Group Performance Score, *HR* hazard ratio, *PVI* portal vein invasion

Multivariate Cox regression analysis confirmed independent prognostic value of high gadoxetic acid uptake (HR, 1.7 [95% CI, 1.21–2.3]; *p *= 0.002), Child–Pugh grade B (HR, 2.8 [95% CI, 1.75–2.6]; *p* < 0.001), larger tumor diameter (HR, 1.6 [95% CI, 1.18–2.1]; *p* = 0.002), and ALBI grade 2 (HR, 1.6 [95% CI, 1.25–2.1]; *p* < 0.001).

Considering only the patients with high gadoxetic acid uptake (*n* = 51), 17 patients received combination therapy (sorafenib and SIRT), and 34 were treated with sorafenib monotherapy. Despite the lack of statistical significance, patients who received combination therapy had longer survival than patients who received sorafenib alone (12.0 vs. 8.89 months, *p* = 0.38; supplementary Fig. 1).

Follow-up images were available in 231 (74.0%) patients. Despite the lack of statistical significance, patients with high gadoxetic acid uptake had shorter progression-free survival compared to patients with low gadoxetic acid uptake (5.9 vs. 6.4 months, *p* = 0.41). Similarly, considering treatment arms separately, patients with high gadoxetic acid uptake had shorter progression-free survival in the combination arm (6.0 vs. 7.8 months, *p* = 0.58) and the sorafenib arm (4.5 vs. 5.95 months, *p* = 0.61).

## Discussion

Our results show that high gadoxetic acid uptake of the tumor is associated with shorter overall survival in HCC patients who received sorafenib. There was no significant association between gadoxetic acid uptake and tumor burden or liver function status of the patient. High gadoxetic acid uptake of the tumor remained an independent prognostic factor for overall survival in the multivariate analysis with other established prognostic factors, including Child–Pugh stage, tumor diameter, and ALBI grade.

Gadoxetic acid is a hepatocyte-specific gadolinium-based contrast media taken up by hepatocytes starting in the transitional phase via organic anion transporting polypeptides (OATP). Although HCC lesions typically are hypointense on the hepatobiliary phase (missing uptake of gadoxetic acid), previous studies have reported up to 10–20% of iso-hyperintensity (preserved or increased uptake of gadoxetic acid) of HCC (Kitao et al. 2010, 2015; Ariizumi et al. 2019). Previous studies have shown a decrease in OATP concentrations of the lesions as the carcinogenesis progresses (Yamashita et al. 2014). A previous study has shown that HCC lesions with gadoxetic acid uptake have overexpression of OATP1B3 compared to lesions with no uptake (Narita et al. 2009), and RTE had a perfect correlation (correlation coefficient of 0.91) with OATP1B3 levels on immunohistochemical staining. Yamashita et al. confirmed these findings in 70 HCC cases using PCR and immunohistochemical analyses (Yamashita et al. 2014). Therefore, the utilization of gadoxetic acid-enhanced MRI has been suggested as an imaging biomarker of the OATP status of the tumor cells. Additionally, it was shown that increased OATP1B3 expression is correlated with WNT signaling. Kitao et al. have shown that HCCs with the ß-catenin mutation have significantly higher RTE than HCCs without (Kitao et al. 2015). This observation was also confirmed with increased expression of OATP1B3 in ß-catenin mutated HCCs. Furthermore, Ueno et al. demonstrated that OATP1B3 mRNA expression is associated with downstream targets of the WNT/ ß-catenin pathway, such as CYP2E1, GS, OAT, AXIN2, and LGR5; and they showed the ratio of RTE/RLE with a cut-off value of 0.9 has 78.9% sensitivity and 81.7% specificity to predict the ß-catenin mutation status of HCC (Ueno et al. 2014). Therefore, gadoxetic acid uptake is a valid surrogate of ß-catenin mutation.

Sorafenib, a multikinase inhibitor whose targets include, amongst others, the RAF/MEK/ERK pathway in tumor cells and tyrosine kinases VEGFR/PDGFR in tumor vasculature, remained the standard of care of advanced HCC for more than a decade following its initial approval in 2007 (Llovet et al. 2008). Since 2017 several treatment options have proven effective in the first and second line, but sorafenib still holds a key role in treatment algorithms and some clinical scoring systems have been described to predict sorafenib benefit (Labeur et al. 2020). Single-agent therapies with immune checkpoint inhibitors have shown disappointing results. Phase III trials for Nivolumab and Pembrolizumab have not reached their prespecified endpoints, after promising results of earlier trials led to approval by the FDA (Yau et al. 2019). A recently proposed immunological classification of HCC separates tumors into three subclasses: Immune, immune intermediate, and immune excluded class (Llovet et al. 2018; Pinyol et al. 2019). HCCs with activated WNT/β-catenin signaling were categorized in the immune exclusion class deemed less likely to respond to immune checkpoint blockade. This is supported by preclinical data. Ruiz de Galarreta et al. utilized a novel mouse model of HCC to show that β-catenin activation promotes immune evasion via deficient dendritic cell recruitment and T-cell activity (“cold tumor”), which eventually led to resistance to anti-PD-1 agents (Ruiz de Galarreta et al. 2019).

Clinical data on this matter are only scarcely available. Harding et al. assessed the potential value of molecular profiling of patients with hepatocellular carcinoma in the treatment decision-making process using next-generation sequencing (Harding et al. 2019). While in the immune checkpoint inhibitor cohort (84% of the patients receiving monotherapy), WNT/β-catenin mutation was associated with poorer PFS, no significant difference in PFS and overall survival was identified in the sorafenib cohort. Considering these results, it has been suggested to use β-catenin mutation status to allocate patients with HCC to treatment with tyrosine kinase inhibitors (TKI) instead of immune checkpoint inhibitors and use gadoxetic acid-enhanced MRI as a potential imaging marker of β-catenin signaling (Kudo 2020a). However, even though the difference was not statistically significant (*p* = 0.15), the overall survival of patients with WNT mutation in the sorafenib cohort was approximately eight months shorter than patients with wild-type WNT (12 vs. 19,96 months). Lack of significance might be the result of relatively small sample size (*n* = 81). Several preclinical studies have identified various molecular pathways related to sorafenib resistance by activating WNT/β-catenin signaling (Lin et al. 2016; Xu et al. 2020), and inhibition of the WNT/β-catenin pathway has been shown to improve the antitumor effect of sorafenib (Muche et al. 2014; Huang et al. 2017). Our results are in agreement with these preclinical studies. High gadoxetic acid uptake of the tumor, a surrogate of the activation of the β-catenin pathway, was associated with poorer overall survival in patients with HCC who received sorafenib. Considering these studies together with our results suggests patients with WNT/β-catenin mutation could be resistant to sorafenib therapy, in addition to immune checkpoint inhibitors, and additional therapeutic measures might be needed in these patients. Although the combination of atezolizumab (an anti-PD-L1 antibody) and bevacizumab (VEGF inhibitor) improved the median overall survival to 19.2 months compared to 13.4 months with sorafenib (Finn et al. 2020, 2021), it remains unclear if combination therapies can overcome the limitations of single-agent approaches in WNT/β-catenin mutated tumors proposed above. Increased response and lower rates of progressive disease seen in clinical trials published to date seem to point in that direction, and there is also a pathophysiological rationale supporting this assumption (Kudo 2020a; Lee et al. 2020). For example, in a trial evaluating the combination of Lenvatinib and Nivolumab, the progressive disease rate was lower than the fraction expected to be of the immune exclusion class histology (Kudo et al. 2020b).

Contradictory to the conclusions of Harding et al. mentioned earlier, our findings suggest, using an imaging-based surrogate, ß-catenin activation support resistance to sorafenib treatment. To elucidate this further, many questions remain to be answered. For example, in the combination arm of the SORAMIC trial, patients received SIRT in addition to sorafenib. When only the patients with high gadoxetic acid uptake were considered, there was some improvement in survival, although the difference was statistically non-significant, probably due to the low number of patients. Furthermore, in addition to validating our results in a sorafenib treated cohort, in which the ß-catenin mutation status was proven by histopathological examination or next-generation sequencing, the role of other TKI agents in ß-catenin mutated population also needs to be evaluated. Lenvatinib, another TKI that was proven non-inferior to sorafenib as a single agent and has shown promise in combination with anti-PD-1 antibodies, is of particular interest in this regard (Kudo et al. 2018; Kudo et al. 2020b).

This study has some limitations. First, baseline images were missing for 12.1% of the patients. However, it is still acceptable in a European multicenter trial and it was due to major protocol deviations. Second, only the index lesion was evaluated and tumor tissues were not available for pathological evaluation of the mutation status of the patients. However, in 89.9% of the lesions, the index lesion and second-largest lesion had the same imaging appearance compared to adjacent liver parenchyma (low vs. iso/high intensity, data not shown). Finally, due to missing imaging in a considerable number of cases, despite the lower progression-free survival in patients with high gadoxetic acid uptake in all cohort, as well as both treatment groups, we were not able to identify the statistical significance. But overall survival is unbiased in our cohort due to lack of effective therapies applied beyond progression. During the trial period, there were no approved second-line therapies; only 17 patients received further anticancer therapies, of whom only seven had drugs that were approved in the future (Supplementary Table 1). Considering the low number of patients, we believe this situation has no significant effect on the overall survival of the cohort. Furthermore, this study presents the first cohort in the literature evaluating the prognostic value of gadoxetic acid uptake in 312 HCC patients receiving sorafenib treatment within a multinational prospective trial, in which all patients underwent imaging with a standardized protocol and all had same image sequence technique for measurements (3D GRE). In our study, quantitative measurements (RTE/RLE) were used to identify tumors with high contrast uptake instead of visual assessment of signal intensity, as done in most previous studies (Ueno et al. 2014; Kitao et al. 2015), which eliminates the biases related to subjective nature of the visual assessment, as well as the appearance of tumor on pre-contrast images. Furthermore, the cut-off value for RTE/RLE was chosen as 1 in our study to increase specificity as compared to 0.9 from the paper of Ueno et al. (Ueno et al. 2014).

In conclusion, our study has shown that high gadoxetic acid uptake of HCC lesions, which is a potential surrogate of ß-catenin activation, is prognostic for worse overall survival in patients who received sorafenib. After validation of this result in larger cohorts, gadoxetic acid-enhanced MRI could serve to identify patients with treatment resistance and be used in the treatment allocation and decision-making process of patients with HCC.

## Supplementary Information

Below is the link to the electronic supplementary material.

Supplementary file1 (PNG 132 KB) Supplementary Fig. 1. Kaplan Meier curve showing the overall survival of the patients with high gadoxetic acid uptake grouped by the treatment

Supplementary file2 (DOCX 18 KB)

## Data Availability

Data are available through corresponding author upon reasonable request.
