# Supplementary figures and images for: Gadoxetic acid uptake as a molecular imaging biomarker for sorafenib resistance in patients with hepatocellular carcinoma: a post hoc analysis of the SORAMIC trial
Source: J Cancer Res Clin Oncol. 2021 Sep 20;148(9):2487–96. doi: 10.1007/s00432-021-03803-3 (PMC9349099; doi:10.1007/s00432-021-03803-3)

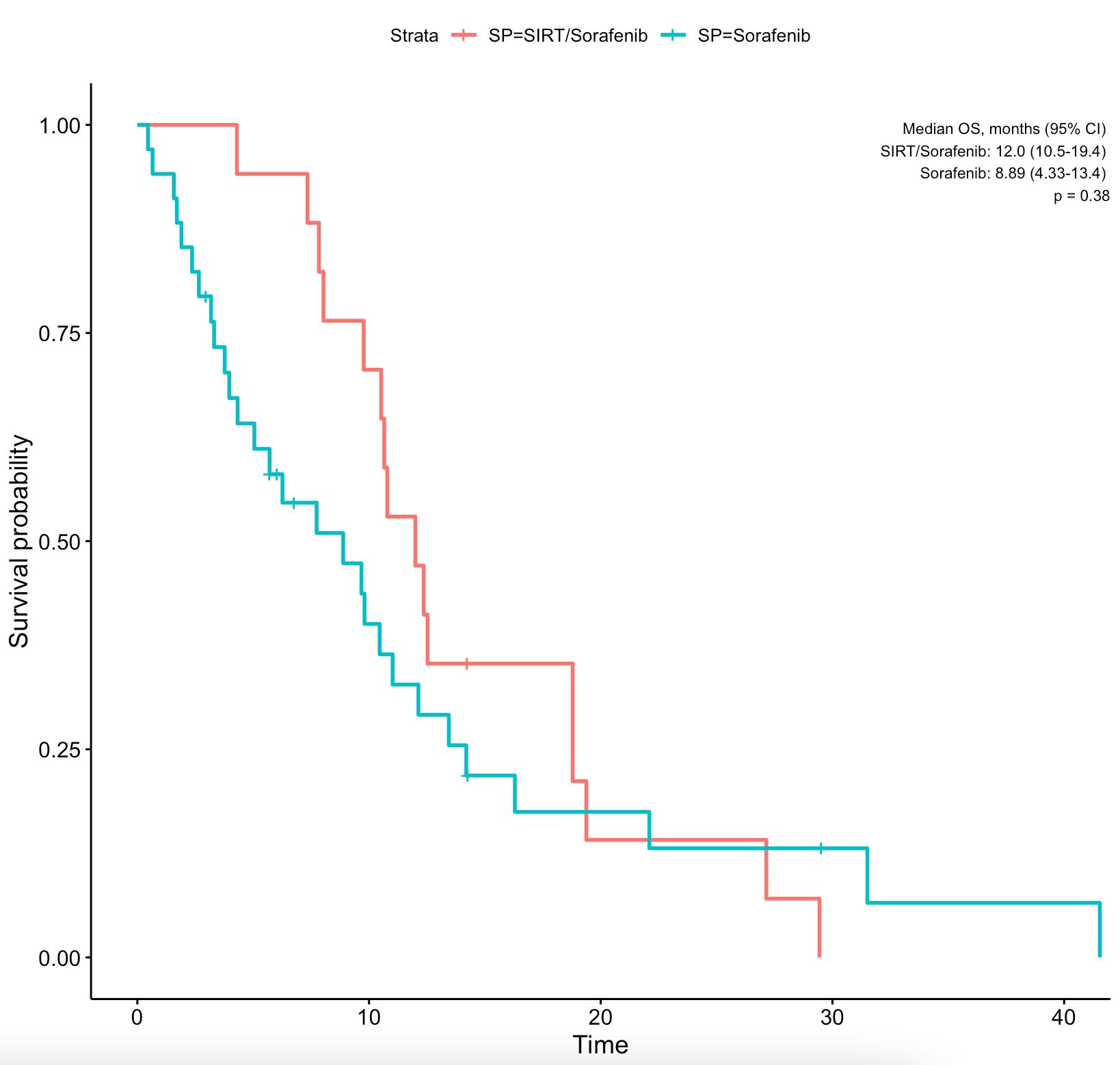

Supplement: Supplementary file 1 — Supplementary file1 (PNG 132 KB) Supplementary Fig. 1. Kaplan Meier curve showing the overall survival of the patients with high gadoxetic acid uptake grouped by the treatment [file 432_2021_3803_MOESM1_ESM.png]
