# Supplementary material for: Gadoxetic acid uptake as a molecular imaging biomarker for sorafenib resistance in patients with hepatocellular carcinoma: a post hoc analysis of the SORAMIC trial
Source: J Cancer Res Clin Oncol. 2021 Sep 20;148(9):2487–96. doi: 10.1007/s00432-021-03803-3 (PMC9349099; doi:10.1007/s00432-021-03803-3)
Supplement: Supplementary file 2 — Supplementary file2 (DOCX 18 KB) [file 432_2021_3803_MOESM2_ESM.docx]

**Supplementary table 1. Anticancer therapies beyond trial treatment**

| Patient | Drug / Treatment | Reason |
| --- | --- | --- |
| 1 | TACE | HCC progression |
| 2 | Gemcitabine, Oxaliplatin, Everolimus | HCC progression |
| **3** | **Fluorouracil** | **Skin cancer** |
| 4 | Cabozantinib | HCC progression |
| 5 | Cabozantinib | HCC progression |
| 6 | Cabozantinib | HCC progression |
| 7 | Cabozantinib | HCC progression |
| 8 | Gemcitabine, Cisplatin | HCC progression |
| 9 | Cabozantinib, Oxaliplatin, Fluorouracil, Folinic acid | HCC progression |
| **10** | **Jx 594** | **HCC progression** |
| 11 | Tasquinimod | HCC progression |
| 12 | Tasquinimod, Gemcitabine, Oxaliplatin | HCC progression |
| **13** | **Cabozantinib** | **HCC progression** |
| 14 | Regorafenib | HCC progression |
| 15 | Gemcitabine, Oxaliplatin, Everolimus | HCC progression |
| 16 | Gemcitabine, Oxaliplatin, Everolimus | HCC progression |
| 17 | Tepotinib / TACE | HCC progression |
| 18 | Tepotinib | HCC progression |
| 19 | Tepotinib | HCC progression |

Bold type indicates patients with high gadoxetic-acid uptake.

HCC: Hepatocellular carcinoma, TACE: Transarterial chemoembolization
